# Procedural Curriculum to Verify Intern Competence Prior to Patient Care

**DOI:** 10.5811/westjem.2022.11.58057

**Published:** 2022-12-29

**Authors:** Jennifer Yee, Christopher San Miguel, Sorabh Khandelwal, David P. Way, Ashish R. Panchal

**Affiliations:** The Ohio State University College of Medicine, Department of Emergency Medicine, Columbus, Ohio

## Abstract

**Introduction:**

Emergency medicine (EM) programs train residents to perform clinical procedures with known iatrogenic risks. Currently, there is no established framework for graduating medical students to demonstrate procedural competency prior to matriculating into residency. Mastery-based learning has demonstrated improved patient-safety outcomes. Incorporation of this framework allows learners to demonstrate procedural competency to a predetermined standard in the simulation laboratory prior to performing invasive procedures on patients in the clinical setting. This study describes the creation and implementation of a competency-based procedural curriculum for first-year EM residents using simulation to prepare learners for supervised participation in procedures during patient care.

**Methods:**

Checklists were developed internally for five high-risk procedures (central venous line placement, endotracheal intubation, lumbar puncture, paracentesis, chest tube placement). Performance standards were developed using Mastery-Angoff methods. Minimum passing scores were determined for each procedure. Over a two-year period, 38 residents underwent baseline assessment, deliberate practice, and post-testing against the passing standard score to demonstrate procedural competency in the simulation laboratory during intern orientation.

**Results:**

We found that 37% of residents required more than one attempt to achieve the minimum passing score on some procedures, however, all residents ultimately met the competency standard on all five high-risk procedures in simulation. One critical incident of central venous catheter guideline retention was identified in the simulation laboratory during the second year of implementation.

**Conclusion:**

All incoming first-year EM residents demonstrated procedural competence on five different procedures using a mastery-based educational framework. A competency-based EM curriculum allowed for demonstration of procedural competence prior to resident participation in supervised clinical patient care.

## INTRODUCTION

The transition from medical student to intern is abrupt and filled with challenging new roles and responsibilities. In procedural specialties such as emergency medicine (EM), new interns are expected to perform bedside procedures under the supervision of senior residents and attending physicians. However, students’ experiences with procedures and the degree of supervision provided are significantly variable. Consequently, procedures performed by unprepared clinicians are a significant contributor of medical errors, which are in turn a major source of morbidity and mortality in the United States.^1^ Researchers have estimated that between 13.5–24.5% of all adverse events in the US healthcare system are due to iatrogenic procedural complications.^2^–^4^ In response to this threat to patient safety, there has been a widespread movement to evaluate and address sources of medical error including improvement of the procedural education of trainees.^5^

The Association of American Medical Colleges (AAMC) published Core Entrustable Professional Activities (EPA) to provide expectations for the activities that medical students should be able to perform upon entering residency.^6^ Entrustment was clearly defined as “trustworthiness in applying the requisite knowledge, skills, and attitudes” when performing a clinical activity. Upon entering an EM residency, programs assess their learners with milestones created by the American Board of Emergency Medicine (ABEM) and the Accreditation Council for Graduate Medical Education (ACGME).^7^ Level 1 milestones define skills expected of medical school graduates, which should correspond to the AAMC’s EPAs. However, gaps in skills and knowledge can occur in the handoff between medical school and residency training, particularly when it comes to the ability to perform procedures.^8^ Furthermore, both EPAs and EM milestones describe procedural competency in general terms rather than through specific standards that define competence. Trainees, therefore, enter residency with a myriad of knowledge and experience with procedures they are now expected to perform as residents. This heterogeneity in procedural education prior to residency exposes patients to avoidable risk from trainees who matriculate with unverified competence to perform procedures.

To optimize procedural performance, rigorous training paradigms that support verification of competence are needed. One method for confirming procedural competency is simulation-based mastery learning (SBML), which incorporates baseline skills assessment, engagement in educational activities, deliberate practice with feedback, and testing until a minimum passing standard (MPS) is achieved.^9^ Through this process, learners attain a standard level of performance without limitations on the time needed to achieve that standard.^9^ The purpose of this project was to apply SBML to prepare entering EM learners to perform (under supervision) the most common, high-risk procedures they will be required to implement during training. This program sets the standards for competence and provides the opportunity for residents to demonstrate achievement of those standards on these procedures in simulation, so they can participate in these procedures (under supervision) during training without putting patients at risk.

## METHODS

### Study Design, Setting and Population

This study was a prospective observational study of the implementation and outcomes of a SBML procedural training curriculum. The curriculum was designed and implemented for incoming EM and EM-internal medicine (EM-IM) residents; it was launched during orientation in July 2020 and continued in 2021. The overall goal was to implement a mastery-based procedural training program to enhance training and verify procedural competency in five common, high-risk procedures performed in EM, including ultrasound-guided internal jugular central venous catheter (CVC) placement, endotracheal intubation (ETI), lumbar puncture (LP), paracentesis, and tube thoracostomy (or chest tube, CT). These procedures were chosen because of their inclusion in the ACGME’s Review Committee for Emergency Medicine’s Key Index Procedure list,^10^ documented iatrogenic risk,^11^–^17^ and/or availability of commercial task trainers. The study was deemed exempt by our institutional internal review board (IRB #2020E0236).

**Population Health Research Capsule**

What do we already know about this issue?

*Iatrogenic procedural injury is a known cause of patient harm. Simulation-based Mastery Learning (SBML) methods for procedures has demonstrated improved patient outcomes.*

What was the research question?

*Would an SBML curriculum enable EM interns to attain and demonstrate procedural competence during orientation?*

What was the major finding of the study?

*All interns achieved competence on five high-risk procedures with improvement from pre- to post-scores (p<0.05).*

How does this improve population health?

*SBML served as a method for verifying the procedural competence of EM interns prior to their involvement with actual patient procedures.*

**Figure 1:**
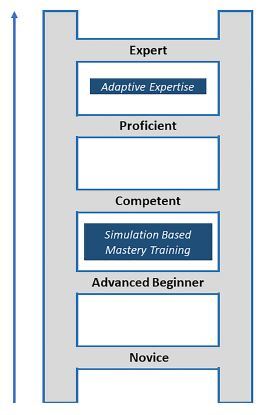
Model depicting progression of procedural skill development from novice to expert. Simulation-based mastery training helps prepare trainees to enter the clinical care environment at a competent skill level without risk to patients. In contrast, adaptive expertise is essential to move to high proficiency and expert levels. This is the ability to understand and manage complex patients and situational conditions thus achieving the optimal patient outcomes.^18^

We designed this program from the perspective of Griswold-Theodorson’s synthesis of the Dreyfus/Benner SBML model of skill acquisition.^18^ Using this model, we introduced SBML to matriculating residents with the assumption that they had already progressed through the “novice” stage and were in the “advanced beginner” stage of development.^18^ Advanced beginners are characterized as having early working knowledge of key aspects of a technical skill and view them as interrelated steps; however, they still require guidance for successful procedure completion.^18^ The goal of our program was to implement SBML to move our matriculating interns from the “advanced beginner” stage to the level of “competent.”^18^ The competent intern is able to safely participate in performing procedures during patient care encounters with gradually decreasing supervision as they move from competent to proficient. (See Figure 1.)^18^

The setting was a university-based, tertiary-care teaching hospital and its three-year EM program with 17 residents per year and a five-year EM-IM residency program with two residents per year. As part of the project development plan, mastery-based training paradigms were developed following commonly described methodology that included the following: 1) development of procedural assessment checklists and standard setting to define competency and 2) mastery training implementation (pretesting and deliberate practice) followed by 3) post-testing to confirm achievement of competency for supervised clinical care.^9^

### Assessment Checklist and Standard Setting

Assessment checklists were developed de novo to address institution-specific safety protocols, such as verbalizing when guidewires are inserted or removed. Checklists for the designated procedures were initially developed by a panel of 13 EM faculty members. These faculty were chosen for their varied expertise with procedures, procedural education, and program administration (Appendix 1). Checklists were developed with each item being scored dichotomously (correct or incorrect) and each item being given equal weight for scoring. Skills assessed by the checklists included maintaining sterility, ultrasound competency, and universal safety items such as calling for time-outs. Consensus on checklist items was achieved through an iterative process of addition and deletion of individual procedural microtasks.

Performance standards were set for these internally developed checklist instruments using methods previously described.^19^ In brief, faculty panelists applied the Mastery-Angoff method by assigning a percentage to each checklist item that represented how many well-trained residents would be able to perform that step correctly without bedside supervision.^20^ To evaluate the overall performance of each procedural checklist, internal consistency reliability among items was estimated using Cronbach’s alpha. For each panelist, the scores of each checklist item were added up for a total raw procedure score. The raw score was then divided by the number of checklist items to determine the MPS specific to each checklist. The MPS was then averaged between all the panelists’ scores for the final MPS for each procedure. During the first year of implementation, we evaluated the item-total correlations (point-biserial correlations) of each item on each checklist. Items with point-biserial correlations less than or equal to .20 (r_ptbis_≤.20) were dropped from further use.

### Mastery Training Implementation

**Figure 2:**
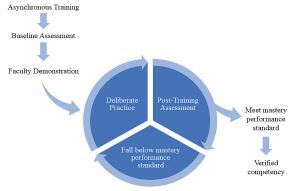
Mastery-based curriculum structure. Included in the curriculum is baseline assessment, deliberate practice with feedback, post-training assessment for competence attainment. If minimum performance standard (MPS) is achieved, the trainee has verified competence. If MPS is not achieved, the trainee continues deliberate practice with feedback until prepared to reassess.

The SBML training was used to instruct incoming first-year EM and EM-IM residents in procedural skills and to confirm achievement of competence.^21^ This included asynchronous pre-training with videos followed by a face-to-face training event (Figure 2). These videos, which included the *New England Journal of Medicine* procedure series (Year One), were assigned prior to the resident’s scheduled time in the simulation laboratory.^22^–^26^ Internally created videos that specifically covered the content of all procedural checklist content were used in Year Two. The internally created videos included institution-specific patient safety initiatives, such as verbalizing when guidewires are inserted and when they are removed, as well as a review of the commercial kit contents used by our institution.

Participants were then provided face-to-face training including pretesting assessments, faculty demonstration, time for deliberate practice, and a post-test to confirm the achievement of competence. This experience was facilitated by faculty who were trained to teach the five procedures and score the checklist assessments. During training, faculty were specifically instructed in how to score each item in the checklists and were instructed not to provide prompting or coaching during pre- or post-testing unless explicitly told to do so on their checklist.

Face-to-face skills training events were held over three days to accommodate all interns. Sufficient time was designated to allow for pretesting, faculty demonstration, deliberate practice time, and post-testing. Included in the overall training period was additional time accommodations for repeat education, deliberate practice, and post-testing if competency was not achieved. One faculty member was assigned to each participant for the day. During pretesting, checklists were used to obtain participants’ individual baseline performance on the procedures (up to 30-minute pretesting period).

Each participant’s performance was compared against the MPS derived from standard setting. Afterward, a faculty member then demonstrated the procedures for the group of participants. Participants were able to ask questions and were given clarity on critical procedural steps. Following this, each participant then returned to their assigned faculty member for deliberate practice and receipt of individualized feedback. Participants were given up to 45 minutes of deliberate practice per procedure. At a minimum, the individual participant’s time commitment for all five procedures was estimated at nine hours if they were able to pass each post-test on their first attempt after one session of deliberate practice.

### Post-testing for Competence Verification

Deliberate practice was followed by post-testing to assess whether procedural competency was achieved. If the participant did not achieve the MPS on their first attempt, they were assigned to additional deliberate practice until they were ready for a repeat post-test. This was repeated for each time the MPS was not achieved by the participant as outlined by mastery-learning principles to train everyone to a preset standard, resulting in outcomes with minimal variation. During the second year, we added the identification of critical incidents or “dangerous actions,” such as a retained guidewire, dilation of a carotid artery, or a CVC advanced in a cephalad direction that were likely to lead to patient harm. As they were thought to be critical enough to be identified and remediated immediately, if these actions were identified during pretesting or post-testing, the facilitator would immediately stop the attempt and debrief with the participant. Participants were then directed to additional deliberate practice to optimize skills.

### Analysis

Descriptive statistics were calculated for demographic characteristics of the population. We used cumulative percentages (with frequencies) to describe achievement of competence, per assessment attempt, for each procedure. Box and whisker plots were generated to demonstrate the percent correct per procedure for baseline and final comparisons. These plots described the median, interquartile ranges, and upper and lower extremes. Outliers are demonstrated by single points below or above the plot. We used Wilcoxon matched-pairs signed-rank tests to evaluate differences between baseline and final assessment scores. All analyses were completed using Stata SE version 17 (StataCorp LP, College Station, TX).

## RESULTS

In 2020 and 2021, 38 matriculating EM and EM-IM residents were enrolled in the training program. These residents were 47.4% women with a median age of 27.5 years. This group had a higher composition of women than the national average for EM residents in the United States, which was 36.9% female in 2020–2021.^27^

**Figure 3:**
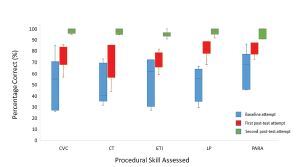
Improvement in performance on procedures for learners requiring greater than one post-test assessment: CVC (n=6), CT (n=6), ETI (n−=6), LP (n=6) and para (n=7). *CVC*, central venous catheter; *CT*, chest tube; *ETI*, endotracheal intubation; *LP*, lumbar puncture; *Para*, paracentesis.

Greater than 90% of participants were able to achieve the MPS on all five procedures within two attempts after baseline assessment (Table 1). All participants achieved competence by the third attempt. A total of 14 participants needed more than one attempt at post-testing (37%), including six participants for CVC placement, six for CT, six for ETI, six for LP, and seven for paracentesis. Five learners (13.16%) needed more than one attempt on one procedure, five learners needed more than one attempt on two procedures, two learners (5.26%) for three procedures, and two learners for all five procedures. Twenty-four participants (63%) were able to demonstrate competency in all five procedures after a single session of instruction and deliberate practice. The learning pattern for participants who required more than one attempt is noted in Figure 3. As expected through the mastery-based training platform, performance improved to eventual attainment of competence in all procedures for learners who required additional attempts.

**Figure 4:**
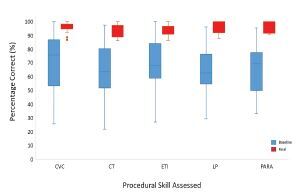
Improvement from baseline to final assessment in the 5 procedures (n=38). Box whisker plot with median and interquartile range; *, P<0.05. *CVC*, central venous catheter; *CT*, chest tube; *ETI*, endotracheal intubation; *LP*, lumbar puncture; *Para*, paracentesis.

There was a significant improvement in performance for each procedure, from baseline to final post-assessment, for the whole cohort as noted in Figure 4 (*P*≤0.05, baseline testing to final post-assessment scores). Additionally, the extent of variability in scores significantly decreased from baseline to final post-assessment.

Total time for attainment of competence for participants was tracked through the study. Total time per participant, if the MPS was achieved on all five procedures after one post-test attempt, was approximately nine hours. The maximum amount of time required by a participant to achieve competency on all procedures was 16 hours. Of note, dangerous actions, tracked in year two, were rare with only one event occurring where a guidewire was retained during post-testing.

## DISCUSSION

In an ideal world, all EM trainees would be supervised throughout the entirety of a clinical procedure. In reality, however, faculty step away from the bedside to care for another sick patient, become distracted with a phone call, or may not have training themselves on how to troubleshoot learners who encounter procedural difficulties. Through this mastery-based learning program, all participants achieved a level of procedural competence, as defined by preset standards, in the simulation laboratory. This cleared residents for supervised participation in procedures during clinical care, ameliorating the risk of novice iatrogenic injuries that result from “practicing” on a patient.

One significant observation of this study is that 37% of participants required additional deliberate practice and additional attempts to pass the MPS for at least one procedure. This indicated that achievement of skills competency required additional focused deliberate practice and feedback for several interns with a small group needing multiple attempts on several procedures.

Procedural competence is a core aspect of EM training and has been noted to be essential for independent practice.^7^,^10^ Unfortunately, many EM procedures occur at low frequencies,^28^–^32^ and may be associated with high-risk complications.^33^ Key index procedure numbers coupled with procedure logs have been traditionally used by residency programs as indicators of procedural experience or mastery. However, these guidelines have never been supported by any specific literature or validated against competency-based performance assessments. Clear definitions of procedural competence during residency training have not been established, nor has there been a standard framework for how procedural competency should be demonstrated during EM residency training.

Realistically, it is not feasible to assess individual clinicians’ competence regularly to determine their current procedural skillset during residency, nor are procedural competencies reviewed at the level of program accreditation. Mastery-based procedural education has been demonstrated to improve patient outcomes in a variety of settings.^34^ To avoid iatrogenic harm from matriculating first-year residents, the least a program can do is to assess their residents’ procedural competency prior to allowing them to encounter patients in the clinical setting. If incoming residents are lacking procedural competence, training them up to preestablished standards in the simulation lab prepares them to participate in procedures during patient encounters. The next step will be to assess for maintenance of skill over time and translational patient outcomes.

There is currently a lack of literature that describes the actual baseline procedural competency of matriculating EM interns. Our data showed significant variability of baseline performance across five high-risk procedures, suggesting the need for competency-based procedural education at the onset of residency training. While the extent of variability of procedural competence of matriculating first-year residents is unclear, it is an important consideration for educators as they prepare these physicians for clinical practice.

A key factor involved in this study was the amount of time and manpower needed for a comprehensive residency-wide, mastery-based procedural training program. Due to the substantial time and physical resources required, it is possible that implementation of this program may not be universally feasible. The total procedural training time for all procedures averaged 11 hours per participant. Thirty-two facilitators were needed in year one, which was streamlined to 17 facilitators in year two. Upon reflection, a key implementation consideration is the burden of time and educational infrastructure weighed against the overall training benefit for new residents and potential for improvement in patient outcomes.

Resource-limited programs may find this approach challenging but may consider choosing SBML for a different set of procedures or adjust the time schedule to suit their faculty and resource availability. We suggest that interested programs identify procedures based on risk of potential iatrogenic injury, create institution-specific checklists or modify existing published procedural checklists to include any institution-specific safety elements, and obtain faculty buy-in with scheduling education time well in advance to accommodate the time needed for both preparation and education.

## LIMITATIONS

This was a pilot study performed at one clinical site with experts derived from the faculty of a single residency program to develop checklists and standards and provide the teaching for SBML. One key challenge we faced were the logistics of delivering the necessary education in the face of COVID-19 with limited availability of personal protective equipment and required physical distancing between stations. Future work will evaluate the best method for developing subsequent procedural proficiency as well as optimal training delivery while maintaining the health and safety of patients.

Despite careful training of faculty facilitators in mastery-based instruction and use of the checklist assessments, we must also consider that the use of so many facilitators was a limitation that may have contributed to variation in scoring and teaching practices. Future research will need to evaluate assessments and training to improve interrater reliability between assessors. Finally, our residency program has a strong tradition of ultrasound education, including its use in guiding procedures, and these best-practice skills were emphasized during instruction, which may not be the case at other institutions.

## CONCLUSION

Educators should strive to deliver care to patients that is as safe as possible, including ensuring that residents are competent before they are permitted to participate in procedures associated with high risk of iatrogenic injuries. Mastery-based frameworks for procedural education have demonstrated improved patient outcomes. By assessing incoming first-year residents’ procedural skills and training them to a standard, potentially dangerous actions were identified, and procedural competency was able to be demonstrated prior to supervised clinical care.

## Supplementary Information



## Figures and Tables

**Table 1:** Cumulative percentage of participants who achieved competence, by number of post-test attempts, for each procedure (n=38).

	Passed assessment after attempt 1 (frequency, %)	Passed assessment after attempt 2 (frequency, %)	Passed assessment after attempt 3 (frequency, %)
CVC	32/38 (84)	35/38 (92)	38/38 (100)
CT	32/38 (84)	37/38 (97)	38/38 (100)
ETI	32/38 (84)	37/38 (97)	38/38 (100)
LP	32/38 (84)	38/38 (100)	n/a
Para	31/38 (82)	37/38 (97)	38/38 (100)

*CVC*, central venous catheter; *CT*, chest tube; *ETI*, endotracheal intubation; *LP*, lumbar puncture; *Para*, paracentesis.
