# Supplementary material for: Procedural Curriculum to Verify Intern Competence Prior to Patient Care
Source: West J Emerg Med. 2022 Dec 29;24(1):8–14. doi: 10.5811/westjem.2022.11.58057 (PMC9897246; doi:10.5811/westjem.2022.11.58057)
Supplement: Supplementary file 1 [file wjem-24-8-s001.docx]

Appendix 1: Composition of emergency medicine faculty panel for development of procedures assessment checklists and standards of performance.

| Number of faculty per skillset | Description of skillset |
| --- | --- |
| 1 | EM simulation director |
| 5 | Residency program leadership faculty |
| 2 | Medical education fellows (past and current) |
| 1 | Director for the medical school’s Advanced Clinical Track in EM |
| 1 | EM faculty considered to be a general procedural expert |
| 1 | EM faculty published in mastery-based learning |
| 2 | EM faculty involved with departmental administration |
